# Size-dependent cryoprotective effects of curcumin nanoparticles on DNA integrity and motility in cryopreserved Angora buck sperm

**DOI:** 10.3389/fvets.2026.1795345

**Published:** 2026-03-30

**Authors:** Ali Erdem Öztürk, Serpil Sarıözkan, Mustafa Bodu, Yunus Emre Atay, Derya Şahin, Oya Korkmaz, Caner Öztürk, Zeliha Kılınç, Muge Mirioglu, Nimet Temur, İbrahim Yasir Teǧiş, Merve Şanlı, Mustafa Numan Bucak, İsmail Öçsoy

**Affiliations:** 1 Department of Reproduction and Artificial Insemination, Faculty of Veterinary Medicine, Erciyes University, Kayseri, Türkiye; 2 Department of Reproduction and Artificial Insemination, Faculty of Veterinary Medicine, Selcuk University, Konya, Türkiye; 3 Department of Obstetrics and Gynecology, Faculty of Veterinary Medicine, Erciyes University, Kayseri, Türkiye; 4 International Livestock Research and Training Center, Ankara, Türkiye; 5 Department of Histology and Embryology, Faculty of Medicine, Malatya Turgut Özal University, Malatya, Türkiye; 6 Department of Reproduction and Artificial Insemination, Faculty of Veterinary Medicine, Aksaray University, Aksaray, Türkiye; 7 Department of Reproduction and Artificial Insemination, Faculty of Veterinary Medicine, Harran University, Şanlıurfa, Türkiye; 8 Department of Chemistry Technology, Vocational School, Alanya University, Antalya, Türkiye; 9 Department of Analytical Chemistry, Faculty of Pharmacy, Erciyes University, Kayseri, Türkiye

**Keywords:** Angora buck, cryopreservation, curcumin, nanoparticle, sperm

## Abstract

Curcumin is a potent antioxidant and anti-inflammatory agent derived from the *Curcuma longa* plant. However, its low water solubility and limited bioavailability restrict its effectiveness in cryopreservation applications. In this study, curcumin nanoparticles (CNp) were produced in two different sizes (20 and 80 nm) to increase bioavailability, and their effects on the freezing of Angora buck sperm were investigated. Semen was collected a total of six times from five Angora bucks (aged 2–3 years). The collected semen was pooled in equal volumes, diluted with a Tris-based egg yolk diluent, and distributed among the experimental groups. Experimental groups set as: control, CNp 20–1 (20 nm CNp at 1 μg/ml), CNp 20-5 (20 nm CNp at 5 μg/ml), CNp 80–1 (80 nm CNp at 1 μg/ml), and CNp 80-5 (80 nm CNp at 5 μg/ml), and were drawn into 0.25 ml straws and frozen. After thawing, motility, kinematic parameters, plasma membrane and acrosomal integrity (PMAI), mitochondrial membrane potential (MMP), and DNA integrity analyses were performed. The highest motility value was observed in the CNp 20-1 group (*p* < 0.05), but the groups containing 80 nm CNp reduced MMP (*p* < 0.01). There was no statistical difference between groups in PMAI. DNA integrity remained within acceptable limits in all groups and was better preserved compared to the control group (*p* < 0.05). In addition to these findings, dynamic light scattering (DLS) analyses revealed that CNps exhibited increased water-absorbing capacity and aggregation. The hydrodynamic diameters of 20 and 80 nm CNps were found to be 223.3 and 289.3 nm, respectively. The findings indicate that the size of curcumin nanoparticles significantly affects aggregation behavior, motility, and DNA integrity parameters. These results suggest that curcumin nanoparticles with a 20 nm size may offer a more effective strategy for the cryopreservation of Angora buck sperm.

## Graphical Abstract

**Figure:**
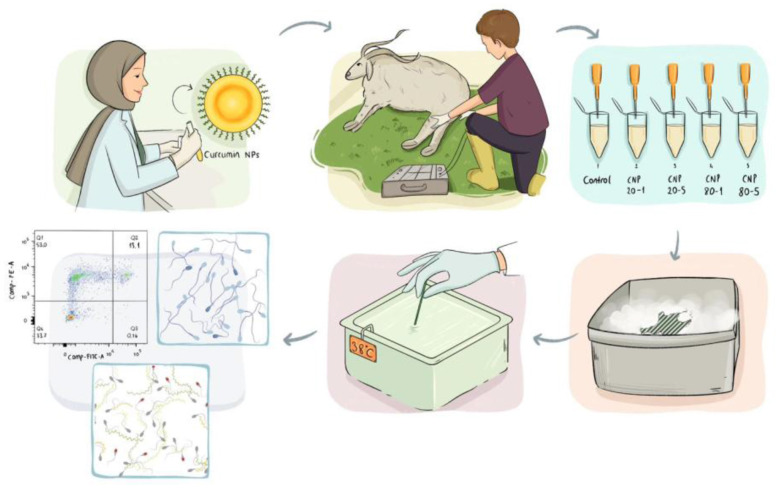
The study design and sequence of the work are indicated by arrows. First, nanomaterials were synthesized, then sperm were collected from bucks by an electro ejaculator and divided into experimental groups. Following dilution, the sperm were frozen in liquid nitrogen vapor. It was then thawed in a 38 °C water bath, and spermatological analyses were performed.

## 1 Introduction

The Angora goat is a breed originating in Ankara, the capital of Türkiye, and is raised throughout the Anatolian region. This breed is recognized for its high mohair yield potential and has also been introduced into breeding programs in countries such as the USA, Argentina, and Southern Africa (1). The number of Angora goats in Türkiye decreased from 1,184,942 in 1991 to 202,243 in 2024 (2). However, in recent years, significant progress has been made toward the preservation of the genetic resources of this species through sperm cryopreservation studies.

However, semen cryopreservation has several limitations, including cold shock and reactive oxygen species (ROS)-induced membrane and DNA damage (3). Increased ROS production during cryopreservation leads to lipid peroxidation in spermatozoa, disruption of mitochondrial membrane potential, and DNA fragmentation (3). In particular, goat spermatozoa, such as the Angora buck, which have a membrane structure containing high levels of polyunsaturated fatty acids (PUFA), are significantly more susceptible to oxidative damage (4). Therefore, antioxidant-enhanced diluents are also important for membrane stability, mitochondrial function, and DNA integrity.

Antioxidants reduce lipid peroxidation in sperm cell membranes by neutralizing reactive oxygen species formed during freezing and thawing. Consequently, antioxidant supplementation contributes to the preservation of post-thaw viability and motility by preserving membrane integrity, mitochondrial function, and DNA stability (5).

Curcumin is a plant-based antioxidant derived from the *Curcuma longa* plant. In previous studies, it has been shown to exhibit anti-inflammatory (6), antioxidant (7), and anticancer (8) properties. However, in studies on cryopreservation of sperm, curcumin scavenges free radicals and prevents lipid peroxidation by increasing the activity of antioxidant enzymes such as superoxide dismutase (SOD), catalase (CAT), and glutathione peroxidase (GSH-PX) (9, 10). This prevents ROS-induced DNA fragmentation during sperm cryopreservation (9–11). Additionally, it has a protective effect on membrane integrity, acrosomal integrity, and motility by stabilizing cell membranes and preserving mitochondrial function (11, 12). Based on these properties, curcumin has been investigated as a protective additive in semen cryopreservation studies (13–15).

However, due to its poor water solubility, its bioavailability is low, and to address this issue, nanoforms have been developed (16–18). Previous studies evaluating curcumin nanoparticles in sperm cryopreservation have reported improvements in total and progressive motility in goat sperm (19), and CNp even demonstrated improved outcomes than standard curcumin in rabbit sperm (13).

Although the use of curcumin nanoparticles in semen cryopreservation has been reported in the current literature, studies systematically comparing biological effects based on particle size are quite limited. In particular, the evaluation of different sizes within the same experimental design and on the same species constitutes a significant gap in literature.

Studies in the literature indicate that as the size of CNps decreases, their solubility in water, bioavailability, and anti-inflammatory activity increase (18, 20). On the other hand, as the particle size increases, their stability decreases, and the tendency for agglomeration rises (21). Upon reviewing the literature on sperm cryopreservation, it becomes apparent that only a single particle size of CNp has been used (13, 22) or the particle size has not been specified at all (23). To fully understand the effects of CNp, conducting sperm cryopreservation studies with different sizes and doses will help fill this gap in the literature. Based on this motivation, in our research, CNp was synthesized in two sizes and used at two doses for the cryopreservation of Angora buck sperm, with spermatological parameters examined.

In this study, it was hypothesized that smaller CNp would exhibit higher bioavailability and that this would lead to a more pronounced improvement in post-thaw spermatological parameters. It has also been predicted that particle size and dose may have size-dependent effects on sperm functions.

## 2 Materials and methods

### 2.1 Reagents

All chemicals and reagents used in the study were obtained from commercial sources. Dichloromethane (34856), citric acid (C0706), glycerol (G2025), fructose (F2543), penicillin–streptomycin–amphotericin B (A5955), FITC-PNA (L7381), toluidine blue (89640), and trisma base (T6066) were purchased from Sigma-Aldrich (St. Louis, MO, USA). Curcumin (81025) was purchased from Cayman Chemical (Ann Arbor, MI, USA). The Live/Dead™ Viability Kit (L7011) and JC-1 dye (T3168) were purchased from Thermo Fisher Scientific (Waltham, MA, USA).

### 2.2 Synthesis of CNp in different sizes and preparing stock solution

The CNPs were prepared using a modified version of the method described in previous literature (16). A hundred mg of curcumin was dissolved in 20 ml of dichloromethane. For 20- and 80-nm CNp, 1 or 3 ml of the solution was added, respectively, to boiling distilled water (18 MΩ) at a flow rate of 0.2 ml/min and subjected to ultrasonication (100 W, 30 kHz). The mixture was incubated until an orange-colored solution was obtained, and then it was stirred at 800 rpm for 20 min at room temperature. Evaporation of the dichloromethane-containing supernatant above the precipitate was carried out at 50 °C in an oven until the whole supernatant evaporated. The principle of adjusting the nanoparticle size was based on varying the solution volume and flow rate (24). The STEM technique was used to analyze the morphology of the synthesized NPs. This process involves bombarding the sample with high-energy electrons and detecting changes in the energies of the scattered electrons to obtain images of the material (25). In this case, the synthesized NPs were dropped onto a copper grid and dried. The structures that adhered to the grid were then imaged using STEM. The FTIR technique was used to characterize the chemical bond structures. FTIR measurements were obtained by placing the prepared pellet in the sample chamber and recording the absorbance spectrum by sending IR radiation within the specified wavelength range (4,000–400 cm^−1^) from the device. The samples used for characterization were not reused for subsequent applications.

**Figure 1:**
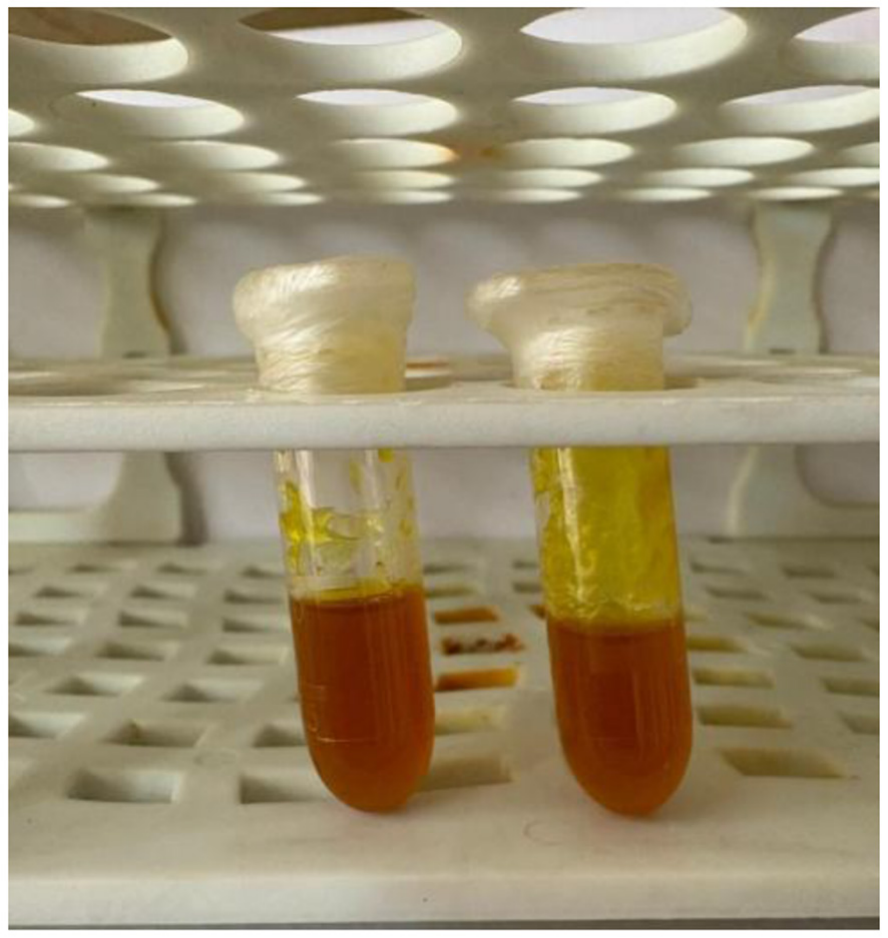
Colloidal dispersion of CNp (20 nm left, 80 nm right) at a dose of 25 mg/ml without forming a precipitate after being kept at 4 °C for several weeks.

CNp stock solutions were prepared as previously described (26). For experimental use, a stock dispersion of CNp was prepared at a concentration of 25 mg/ml. Briefly, 50 mg of CNp was dissolved in 200 μl of dimethyl sulfoxide (DMSO) and subsequently diluted with 1.8 ml of phosphate-buffered saline (PBS) to reach a final volume of 2 ml. The dispersion was vortexed to ensure homogeneity and stored at 4 °C throughout the study. The resulting preparation appeared visually homogeneous, with no observable sedimentation or visible aggregation for either nanoparticle size, as shown in Figure 1.

### 2.3 Animals and semen collection

Five Angora bucks aged 2–3 years that had previously sired offspring were used in the study. Animals were selected randomly from the Angora goat herd at Selcuk University Prof. Dr. Hümeyra Özgen Research and Application Farm. Semen samples were collected with an electroejaculator (P-T Electronics, Model 302, Boring, Oregon, USA) once a week during the breeding season (August–December), resulting in six independent collection sessions. Electrical stimulation was initiated at 2 V and delivered in pulses of 1 s, followed by 1-s rest intervals. Each stimulation set consisted of 10 consecutive pulses. If ejaculation was not achieved after the first set, a second set was applied by increasing the voltage by 1 V. In the second set, stimulation pulses were delivered for 1 s with 2 s rest intervals between pulses (27). Mass sperm activity was assessed according to Evans and Maxwell (28). Briefly, 5 μl of fresh semen was placed onto a glass slide maintained at 37 °C and examined under a phase-contrast microscope at × 40 magnification. The degree of wave motion and coordinated sperm movement was subjectively graded on a scale from 0 to 5, where 0 indicated the absence of movement, and 5 represented very rapid and intense wave-like motion. Mass activity score at least 3, motility of at least 80%, and concentration of 2.5 × 10^9^ were added to the study (28). Semen from five different bucks selected for the study was added to 15 ml centrifuge tubes at a rate of 0.2 ml per animal and mixed. The mixed semen was divided equally among the study groups in a 37 °C water bath.

### 2.4 Experimental design, semen processing, and freezing

The sperm allocated to the experimental groups was diluted using a tris-based egg yolk extender (TRIS, 27.1 g/L Tris, 10.0 g/L fructose, 14.0 g/L citric acid, 20% egg yolk, 5% glycerol, 0.1% penicillin–streptomycin–amphotericin B; pH 6.8, 300 mOsM) served as the primary diluent. Glycerol was incorporated into the extender during its initial preparation, and semen was diluted directly with the final glycerol-containing extender without stepwise addition. The primary diluent was pre-warmed in a 37 °C water bath before being mixed with the semen, and CNp was added in the doses to be used in the study. The CNp doses and sizes used in the study were selected based on those that showed positive and negative effects in previous studies (13, 29). The experimental groups are listed in Table 1.

**Table 1:** Experimental groups.

Groups	Content
Control	TRIS extender
CNp 20-1	TRIS + 20 nm sized 1 μg/ml CNp
CNp 20-5	TRIS + 20 nm sized 5 μg/ml CNp
CNp 80-1	TRIS + 80 nm sized 1 μg/ml CNp
CNp 80-5	TRIS + 80 nm sized 5 μg/ml CNp

Control group and semen samples diluted with nanoparticle-supplemented extenders to a concentration of 1 × 10^8^ spermatozoa per ml were equilibrated at +5 °C for 3 h to facilitate membrane phase transitions. After equilibration, semen was filled into 0.25 ml straws within a +5 °C cold-air cabinet, with the open ends sealed using polyvinyl alcohol powder. The prepared straws were stored horizontally at +5 °C on racks and frozen by exposure to liquid nitrogen vapor for 20 min at a height of 4 cm above the liquid nitrogen surface. Finally, the frozen semen was rapidly plunged into liquid nitrogen and stored in a liquid nitrogen tank (30).

### 2.5 Analysis of motility and kinematic values

Three months after the sperm were frozen, semen samples were thawed in a 38 °C water bath for 25 s (31). Then, sperm were transferred into pre-warmed (35 °C) 1.5 ml centrifuge tubes and maintained on a 35 °C heating plate. A 7.5 μl sample of thawed semen was taken and measured in five different fields using a pre-warmed 20 μm deep counting chamber slide (12050/0220, Minitube, Germany) with the computer-assisted semen analysis (CASA) system (AndroVision, Minitube, Germany) (32). CASA configurations were set as follows: i) at least five different fields (40x) and more than 600 spermatozoa for each sperm sample, ii) 75 frames per second, iii) species-specific decision thresholds defined within the AndroVision^®^ system were applied.

The following parameters were measured in the CASA analysis: (1) total motility (%), (2) progressive motility (%), (3) fast progressive motility (%), (4) slow progressive motility (%), (5) kinetic parameters [straight-line velocity (VSL, μm/s), curvilinear velocity (VCL, μm/s), average path velocity (VAP, μm/s), distance straight line (DSL, μm), wobble (WOB, VAP/VCL), linearity (LIN, VSL/VCL), straightness (STR, VSL/VAP)].

Motile cells were defined according to velocity and trajectory thresholds as follows: spermatozoa with VSL < 24 μm/s and VCL < 48 μm/s were considered locally motile, while those with a radius value between 9–90 μm and rotation > 0.7 were considered circularly motile. Progressive motility is determined based on VCL thresholds, with VCL < 120 μm/s indicating slow progressive motility and VCL ≥ 120 μm/s indicating fast progressive motility. Total motility encompasses all spermatozoa with progressive and non-progressive motility (local and circular motility), excluding immotile cells. Total motility, progressive motility, fast progressive motility, slow progressive motility, and kinematic parameters were measured.

### 2.6 Flow cytometric analyses

For flow cytometric analysis, the CytoFLEX system (CytoFLEX System B4-R0-V0, Beckman Coulter, USA) was used. Sperm samples were analyzed at a wavelength of 488 nm (50 mW laser power). Filters with wavelengths of 525 (FITC) nm, 585 (PE) nm, and 610 (PI) nm were used for fluorescence detection. After gating to exclude debris and non-sperm events based on forward and side scatter characteristics, at least 6,000 spermatozoa were analyzed for each sample (33). Flow cytometric data obtained from FITC-PNA/PI and JC-1/PI staining were analyzed using CytExpert 2.2 software (Beckman Coulter).

#### 2.6.1 Plasma membrane and acrosomal integrity (PMAI)

FITC-PNA (L7381, Sigma–Aldrich Co.) and propidium iodide (PI, P1304MP, Invitrogen) dyes were used to assess plasma membrane and acrosomal integrity. Semen samples were thawed in a 38 °C water bath for 25 s, then transferred into pre-warmed (35 °C) 1.5 ml centrifuge tubes and maintained on a 35 °C heating plate. A 10 μl aliquot of thawed semen was diluted with 190 μl phosphate-buffered saline (PBS) to reach a final concentration of 5 × 106 sperm/ml. Next, 5 μl FITC-PNA and 3 μl PI were added to the prepared semen, and the samples were incubated in the dark at 38 °C for 30 min (34, 35). After the 30-min incubation period, samples were centrifuged at 1,800 × g for 5 min, and the supernatant was discarded. The pellet was resuspended in phosphate-buffered saline (PBS) and gently mixed. Subsequently, the samples were passed through flow cytometry filter tubes to remove debris and aggregates. The prepared samples were then immediately subjected to flow cytometric analysis using the CytoFLEX system.

Flow cytometric evaluation was performed following sequential gating procedures. Initially, spermatozoa were identified, and debris, cell fragments, and non-sperm events were excluded using forward scatter (FSC) and side scatter (SSC) dot plots. Doublet discrimination was subsequently applied using FSC-A vs. FSC-H parameters to ensure analysis of single spermatozoa. PI fluorescence was detected in the 610 nm channel and was used to determine plasma membrane integrity by distinguishing membrane-intact (PI-negative) from membrane-compromised (PI-positive) spermatozoa. FITC fluorescence (525 nm) was used to assess acrosomal integrity based on FITC-PNA binding patterns. Compensation was established using unstained controls as well as FITC-PNA single-stained and PI single-stained samples to correct spectral overlap between FITC (525 nm) and PI (610 nm) channels. Compensation matrices were generated within the CytExpert software before sample acquisition.

#### 2.6.2 Mitochondrial membrane potential

For mitochondrial membrane potential analysis, samples were diluted to a concentration of 5 × 106 sperm/ml, similar to the PMAI procedure. Then, 10 μl of JC-1 (T3168, Invitrogen, 1.53 mm) and 3 μl of PI were added. Samples were incubated at 38 °C for 30 min in a water bath in a dark environment. Flow cytometric analysis was performed after sequential gating steps. Initially, spermatozoa were identified, and debris, cell fragments, and non-sperm events were excluded using forward scatter (FSC) and side scatter (SSC) dot plots. Doublet discrimination was subsequently applied using FSC-A vs. FSC-H parameters to ensure analysis of single spermatozoa. PI-negative spermatozoa were then gated in the 610 nm channel to exclude membrane-compromised cells before mitochondrial membrane potential evaluation. Mitochondrial membrane potential was evaluated based on JC-1 fluorescence distribution. For compensation, unstained controls as well as JC-1 single-stained and PI single-stained samples were used to correct spectral overlap among FITC (525 nm), PE (585 nm), and PI (610 nm) channels. Compensation matrices were calculated within the CytExpert software before sample acquisition. Spermatozoa exhibiting high red/orange fluorescence (JC-1 aggregates) were classified as high mitochondrial membrane potential (HMMP), whereas those showing pre-dominant green fluorescence (JC-1 monomers) were classified as low mitochondrial membrane potential (LMMP), in accordance with the method described by Bollwein, Fuchs (36).

#### 2.6.3 Sperm chromatin condensation

TB staining was performed according to Talebi et al. (2012) with some modifications (37). Five microliter of sperm/PBS mixture was applied to slides and air-dried for 30 min. Air-dried slides were fixed in acetone at 4 °C for 1 h. The slides were then hydrolyzed in 1 N HCl for 15 min at 4 °C, followed by three washes with distilled water for 2 min each. The slides were then stained with 0.05% toluidine blue (pH 4.5) for 10 min at room temperature, rinsed three times with distilled water for 2 min each, and air-dried. For each slide, at least 200 spermatozoa were counted at random in 10 distinct fields using a light microscope (Leica DM2000) and classified as regular TB (+) or aberrant TB (–). Sperm cell heads with high chromatin integrity were light blue, while those with low integrity were dark violet (purple). Toluidine blue staining (TB) interacts with the phosphate groups of DNA strands to reveal the state of sperm chromatin condensation and the degree of DNA fragmentation (38).

### 2.7 Statistical analyses

SPSS 26.0 software was employed to analyze the results. Five ejaculates (one from each animal) were pooled for each replicate, and a total of six independent freezing replicates were performed. Each freezing replicate was considered an experimental unit (*n* = 6). Data normality was checked with the Shapiro–Wilk test and histogram graphs, while homogeneity was assessed using Levene's test. The means of post-thaw spermatological parameters and imaging analysis results were calculated for each experimental group via one-way ANOVA. Groups that showed significant differences within the same parameters were compared using Duncan's multiple-comparison test. Statistical significance was set at *p* < 0.05.

## 3 Results

### 3.1 Characterization results of CNp

**Figure 2:**
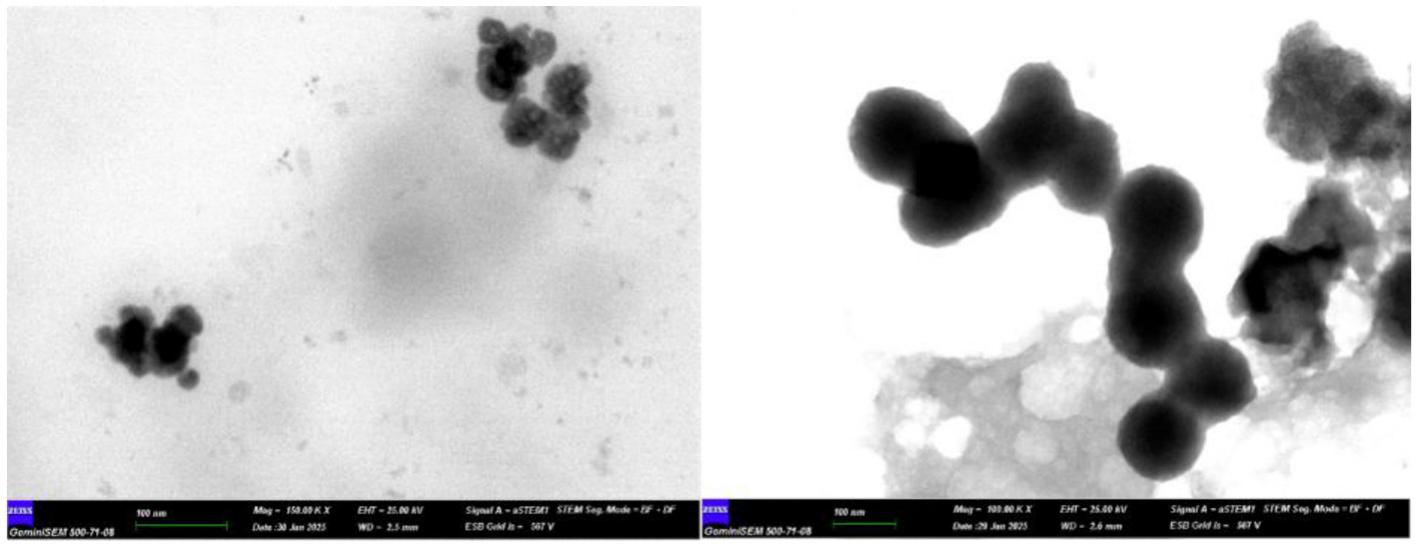
Spherical-shaped Curcumin nanoparticles produced with sizes of 20 nm **(left)** and 80 nm **(right)**. Scale bar: 100 nm.

STEM analysis has demonstrated that CNps with dimensions of approximately 20 and 80 nm have been successfully synthesized. Both CNps exhibit spherical morphology (Figure 2). Particle size measurements were performed in at least five areas, and the observed distribution was found to be consistent with the synthesis targets.

**Figure 3:**
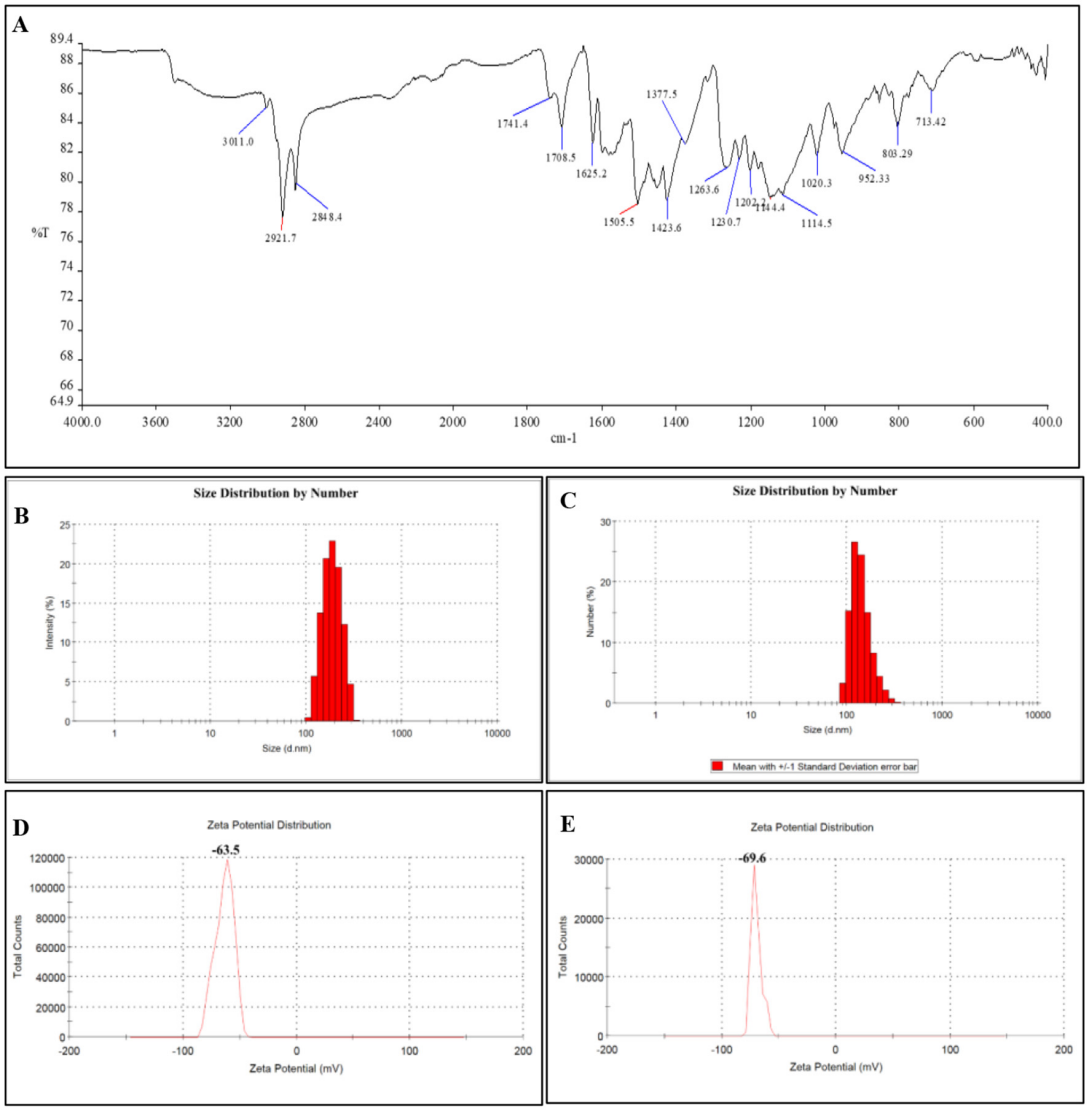
**(A)** FTIR results of 20 nm CNps. Since a different synthesis method was not used to adjust the nanomaterial sizes (solution volume and flow rate were changed), a new FTIR analysis is not required for 80 nm. **(B)** DLS results of 20 nm CNps. The number-weighted size distribution is 192.8 nm, and the hydrodynamic diameter is 223.30 nm. **(C)** DLS results of 80 nm CNps. The number-weighted size distribution is 169.6 nm, and the hydrodynamic diameter is 289.3 nm. **(D)** Zeta potential of 20 nm CNps (−63.5 mV). **(E)** Zeta potential of 80 nm CNps (−69.6 mV).

CNp, regardless of size, exhibited nearly identical FTIR spectrum patterns. The weak stretching peak at about 3,011/cm is attributed to phenolic OH groups. The medium-stretching vibration peak at 2,921/cm can be attributed to C-H groups in alkenes. The carbonyl group (C=O) and an aldehyde group (C–OH) gave medium/weak stretching vibrational peaks at 1,741.4/cm and 1,144.4/cm, respectively. The C=C bonds in alkene gave stretching and bending peaks at around 1,625 and 952/cm, respectively (Figure 3). These data indicate that the chemical structure of curcumin is preserved following nanoparticle synthesis.

When the zeta potential data were evaluated, the 20 nm CNp had a surface charge of −63.5 mV, while the 80 nm CNp had a value of −69.6 mV. The strong negative surface charge of both nanoparticle sizes has been shown to exhibit good colloidal stability.

According to the DLS results, the number-weighted size distribution of 20 nm CNps was 192.8 nm on average, and the Z-average hydrodynamic diameter was 223.3 nm (PDI: 0.322). The number-weighted size distribution of 80 nm CNps was 169.6 nm on average, and the Z-average hydrodynamic diameter was 289.3 nm (PDI: 0.483; Figure 3).

DLS results have shown that both CNp dimensions exhibit a hydrodynamic diameter larger than the primary size in aqueous media. A PDI of 0.322 for 20 nm CNp indicates a relatively narrow distribution, whereas a PDI of 0.483 for 80 nm CNp indicates a broader and more heterogeneous distribution. The fact that the Z-average values are higher than the number-weighted average indicates that the distribution is more sensitive to larger hydrodynamic diameters.

### 3.2 Motility and kinematic values

Upon examining the total motility data, the control group exhibited 38.4% motile spermatozoa. The highest numerical value was observed in the CNp 20-1 group (45.9%), which differed significantly from the control group (*p* < 0.05), while no significant differences were detected among the remaining groups. The total motility rate was 39.8% in both the CNp 20-5 and CNp 80-5 groups, while the CNp 80-1 group had a similar result to the control group with 41.9% total motility (*p* < 0.05).

Progressive motility was determined to be 36.3% in the control group and remained at similar levels in the CNp 20-5 (36.6%), CNp 80-1 (37.2%) and CNp 80-5 (36.6%) groups; in contrast, the CNp 20-1 group (43.5%) was found to be statistically higher than the other groups.

**Figure 4:**
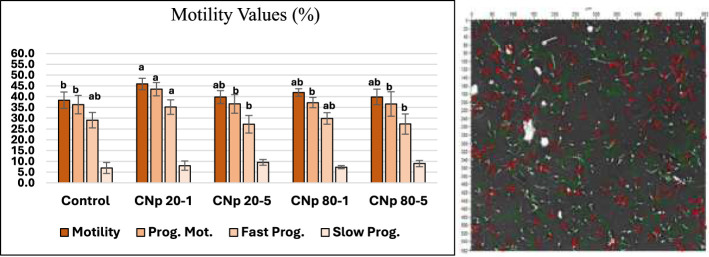
Results of total motility, progressive motility, rapid progressive motility, and slow progressive motility among groups (*p* < 0.05). The group indicated by “a” is statistically similar to the group indicated by “ab”, but significantly higher than the group indicated by “b”. Each parameter (motility, progressive motility, etc.) was statistically compared within its own category. Since no statistical difference was found in the slow progressive motility values, lettering was not applied.

Regarding fast progressive motility, the CNp 20-1 group didn't differ from the control and CNp 80-1 group but was significantly higher than the CNp 20-5 and CNp 80-5 groups (*p* < 0.05). Regarding slow progressive motility, there was no significant difference among the groups (Figure 4).

When the kinematic parameters were evaluated, the control group showed numerically higher values in velocity-related parameters, whereas lower values were observed in the CNp 20-5 group. Although CNp-treated groups tended to present reduced velocity parameters compared with the control group, these differences were not statistically significant (*P* > 0.05). No significant differences were detected among groups for WOB, LIN, and STR parameters (*P* > 0.05; Table 2).

**Table 2:** Kinematic parameter differences among groups (*p* > 0.05).

Groups	VCL (μm/s)	VSL (μm/s)	VAP (μm/s)	DSL (μm)	WOB (VAP/VCL)	LIN (VSL/VCL)	STR (VSL/VAP)
Control	94.44 ± 22.6	38.06 ± 13.08	45.45 ± 14.15	13.75 ± 4.83	0.48 ± 0.05	0.40 ± 0.06	0.84 ± 0.04
CNp 20-1	74.69 ± 8.8	28.77 ± 4.1	35.27 ± 4.45	10.41 ± 2.07	0.47 ± 0.02	0.38 ± 0.03	0.81 ± 0.04
CNp 20-5	66.64 ± 13.5	24.89 ± 6.06	30.76 ± 6.92	8.71 ± 3.12	0.46 ± 0.01	0.37 ± 0.03	0.81 ± 0.03
CNp 80-1	62.75 ± 21.7	23.87 ± 8.03	29.13 ± 9.62	8.50 ± 2.75	0.47 ± 0.03	0.38 ± 0.04	0.82 ± 0.03
CNp 80-5	76.15 ± 11.41	29.81 ± 3.07	35.97 ± 3.80	9.99 ± 1.02	0.48 ± 0.03	0.39 ± 0.02	0.83 ± 0.01

### 3.3 Flow cytometric analyses

**Figure 5:**
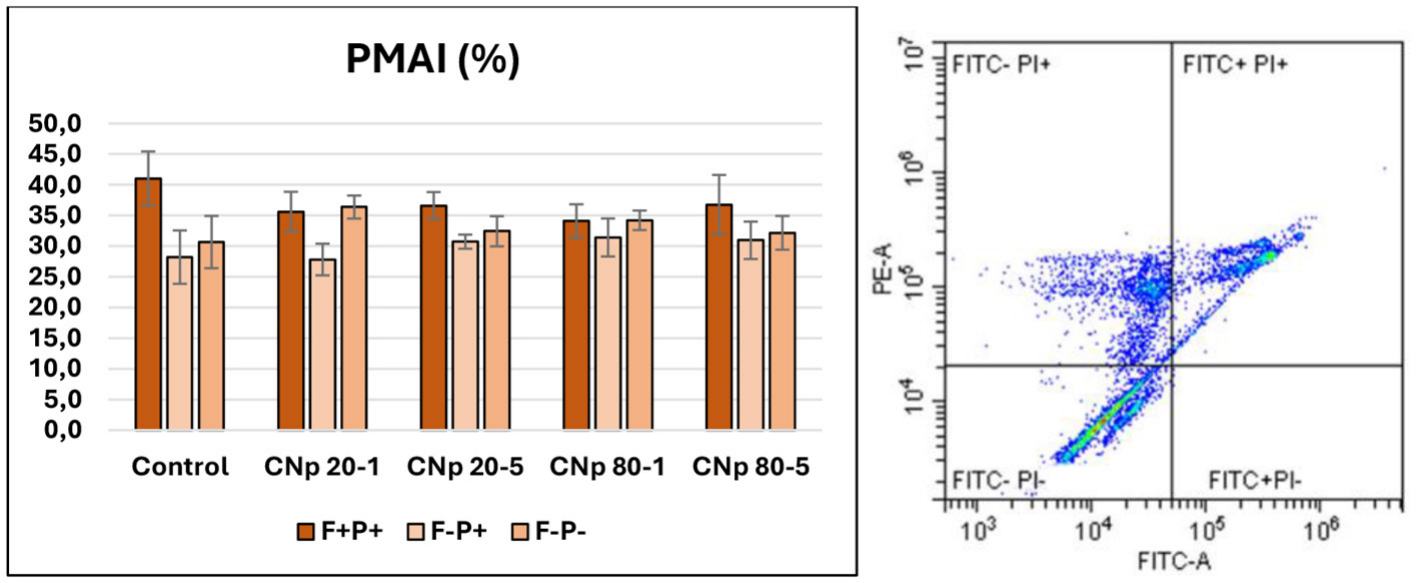
Plasma membrane and acrosomal integrity data. FITC-A (Fluorescein Isothiocyanate Area) and PE-A (Phycoerythrin Area) represent fluorescence intensities in the green and orange-red channels, respectively. FITC-PI– indicates spermatozoa with both intact acrosome and membrane, FITC-PI+ indicates damaged membrane but intact acrosome, FITC+PI+ indicates both damaged acrosome and membrane, and FITC+PI– indicates damaged acrosome and intact membrane (*p* > 0.05).

When evaluating the plasma membrane and acrosomal integrity results, the CNp 20-1 and CNp 80-1 groups showed higher membrane integrity (F–P–) compared to the control and other groups; however, this increase was not statistically significant (*p* > 0.05). Regarding acrosomal integrity (considering the sum of F–P+ and F–P–), all groups demonstrated more favorable results than the control group. Nonetheless, these differences were not statistically significant (*P* > 0.05; Figure 5).

**Figure 6:**
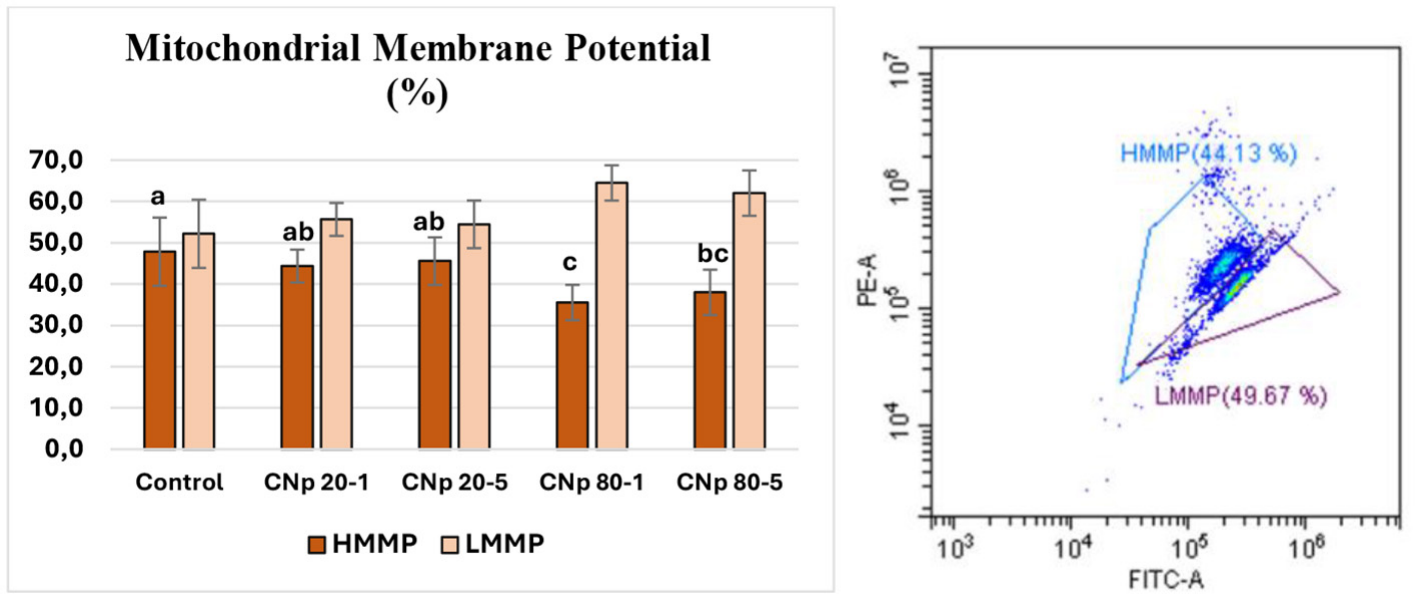
High (HMMP) and low (LMMP) mitochondrial membrane potential results. The group indicated by “a” is statistically similar to the group indicated by “ab” but significantly higher than the groups shown by “b,” “bc,” and “c.” Each parameter (HMMP or LMMP) was statistically compared within its own category. FITC-A (Fluorescein Isothiocyanate Area) and PE-A (Phycoerythrin Area) represent fluorescence intensities in the green and orange-red channels, respectively.

When the mitochondrial membrane potential data were analyzed, the control group and the CNp 20-1 and CNp 20-5 groups showed similar HMMP values. However, the CNp 80-1 and CNp 80-5 groups had significantly lower HMMP values compared to the control group. The LMMP values displayed a pattern opposite that of HMMP, producing similar corresponding results. In other words, mitochondrial function appeared to decline in the groups treated with 80 nm curcumin (*P* < 0.05). However, when comparing with the motility data, the increase in LMMP values did not impact motility, as the groups containing 80 nm CNp showed motility results similar to those of the control group (Figure 6).

### 3.4 Sperm chromatin condensation and DNA integrity

**Figure 7:**
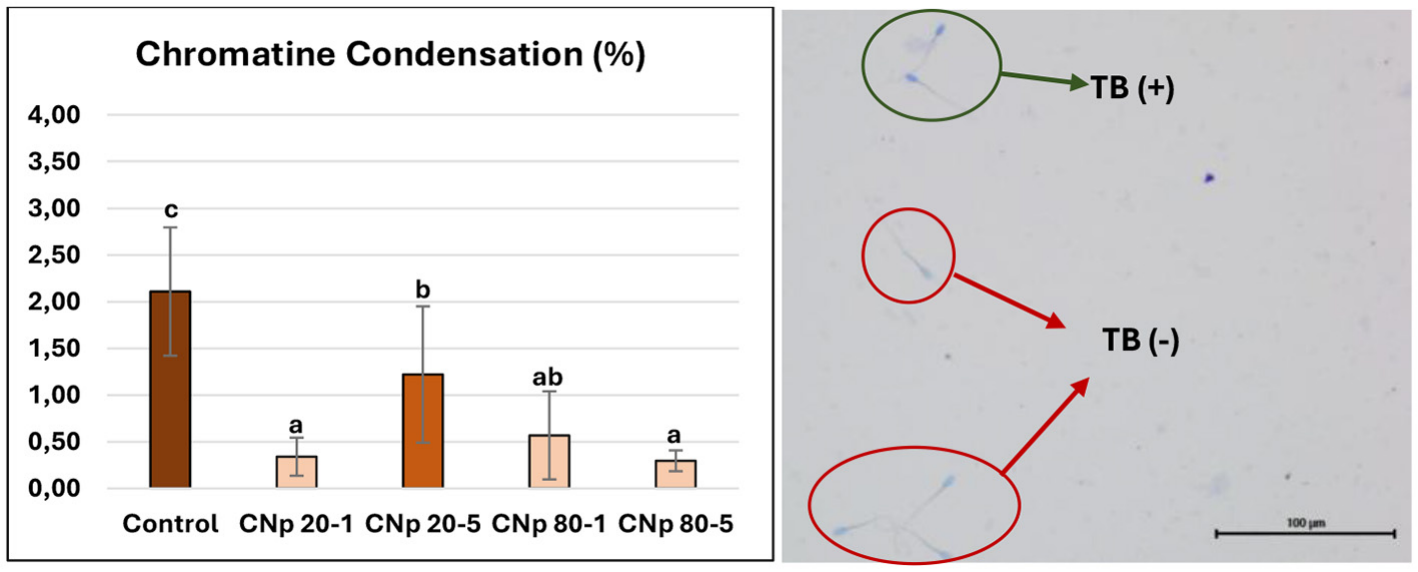
Spermatozoa chromatine condensation rates (*P* < 0.05). The group indicated by “a” has significantly lower DNA damage compared to the group indicated by “b” and “c” but there is no statistically significant difference between the group indicated by “a” and the group indicated by “ab.” The spermatozoa marked with a green circle are stained with toluidine blue and classified as DNA-damaged, whereas the spermatozoa marked with a red circle are unstained and considered undamaged.

According to DNA damage data, DNA damage in the control group was found to be at a level of 2.11%. This rate was lowest in the CNp 80-5 group, at 0.30%. This was followed by the CNp 20-1 (0.34%), CNp 80-1 (0.57%) and CNp 20-5 (1.22%) groups, respectively (Figure 7). Statistically, all groups containing CNp provided protection in DNA integrity compared to the control group, but all values, including those of the control group, were within tolerable limits.

## 4 Discussion

In this study, the effects of CNps of two different doses and sizes on the freezeability of Angora buck sperm were investigated.

When the characterization data of CNPs were evaluated, STEM images showed that the core sizes of CNPs were 20 and 80 nm, respectively. However, DLS analysis revealed that the number-weighted size distribution of 20 nm CNp is 192.8 nm, and the hydrodynamic diameter is 223.3 nm. For 80 nm CNp, the number-weighted size distribution is 169.6 nm, and the hydrodynamic diameter is 289.3 nm.

This discrepancy arises because DLS measures not only the core size but also aggregation and the hydration layer surrounding the core (39). The phenolic and enolic -OH groups on the surface of the CNps form hydrogen bonds with water molecules, creating a hydration layer around the particles, thereby increasing the particle size observed in the DLS measurement (40). Another important point is the aggregation of nanoparticles with an average size of 20 and 80 nm at sizes of 192.8 and 169.6 nm. The reason for the excessive size of nanoparticles in DLS analysis may be their destabilization through Ostwald ripening in an aqueous medium without stabilizers. During this process, smaller particles dissolve due to high resolution and re-accumulate into larger particles, causing the particle size to increase over time and leading to aggregation. Aggregation is observed despite the electrostatic repulsive force, such as a high negative zeta potential (e.g., −69 mV in our study), because Ostwald ripening arises from differences in solubility between particles and is independent of electrostatic interactions (41). This may explain why CNPs with a size of 20 nm exhibit a higher number-weighted size compared to those with a size of 80 nm in our study. Although this mechanism was proposed by Didat et al. (2026), it has not been directly tested in our study.

Considering the spermatological analyses, the most significant increase in total and progressive motility data was observed in the CNp 20-1 group (*p* < 0.05). In contrast, the HMMP ratio was lower in the CNp 80-1 and CNp 80-5 groups (*p* < 0.05). While no significant difference was observed between groups in terms of PMAI data (*p* > 0.05), all groups demonstrated a significant protective effect compared to the control group in terms of chromatin condensation (*p* < 0.05). These findings suggest that CNp application may exhibit size- and dose-dependent biological effects.

Sperm motility is largely dependent on ATP produced in the mitochondria. Increased ROS during cryopreservation leads to disruption of MMP, disruptions in the electron transport chain, and decreased ATP synthesis (3). Once again, increased ROS leads to lipid peroxidation in PUFAs in the membrane, mitochondrial membrane dysfunction, and activation of caspase-modulated apoptosis (42). This cascades results in loss of motility, disruption of membrane integrity and reduced chromatin stability (43).

Oxidative stress disrupts the functioning of the mitochondrial membrane, reducing ATP production and triggering cytochrome c release and caspase activation, thereby initiating the apoptosis process (3). The increase in total and progressive motility observed in the CNp 20-1 group in this study indicates that mitochondrial function and membrane stability were preserved during cryopreservation. Curcumin nanoparticles can regulate mitochondrial membrane function by interrupting free radical chain reactions via phenolic hydroxyl groups (44). This situation may explain the observed increase in motility parameters by contributing to the maintenance of mitochondrial membrane potential and sustained ATP production.

The curcumin molecule, thanks to its lipophilic structure, integrates into the phospholipid layer, thereby inhibiting the peroxidation of unsaturated fatty acids and contributing to the maintenance of membrane fluidity (45). Nanoparticle formulation may help maintain the protective effect at the membrane level by enabling more effective transport of this molecule to the cell surface and increasing its stability (46). In this way, an increase in antioxidative activity has been observed (18, 47). Although there was no statistical difference in PMAI parameters in this study, the observation of a higher intact membrane rate in the groups treated with CNp shows a trend consistent with this mechanism.

The observation of different responses to mitochondrial membrane potential in groups treated with 80 nm particles may be related to size-dependent differences in cellular interactions. Smaller nanoparticles have a higher numerical density and surface area at the same concentration, enabling them to neutralize the ROS to which the cell is exposed more effectively (13, 40). In addition, smaller CNps may exhibit higher endocytosis compared to larger ones (48). Similarly, studies in the literature have shown that smaller CNps (e.g., 7–18 nm) improve motility, plasma membrane, and acrosomal integrity, decrease apoptosis, and enhance fertilization capacity (19, 22). In contrast, the more limited surface area and potential aggregation tendency of 80 nm particles may reduce bioavailability by decreasing their effectiveness in contacting cells. This situation suggests that the more limited functional response observed in the 80 nm groups may be due to differences in biophysical interactions related to particle size rather than direct toxicity. This finding indicates that particle size and dose optimization should be evaluated together in cryopreservation protocols.

A similar protective trend was observed in DNA integrity, with all CNp-containing groups maintaining better DNA integrity than the control group (*p* < 0.05). It is known that curcumin or CNp protects DNA and membrane integrity by interrupting the ROS chain through their phenolic –OH groups (29, 49). The reduction in DNA fragmentation observed in toluidine blue analyses across all CNp groups further supports this mechanism.

In the literature, the effects of CNps on sperm cryopreservation have yielded similar results in different animal species. It has been reported that CNp supports motility, membrane integrity, and mitochondrial function; it also reduces apoptotic processes and oxidative stress markers. This protective effect has been associated with curcumin's ability to scavenge free radicals, support mitochondrial stability, and suppress caspase activation. It is also stated that nanoformulation increases bioavailability, creating a more effective antioxidant defense at the cellular level (13, 19, 22, 23).

The results obtained in our study are highly valuable as they contain findings related to Angora buck semen and present a comparative analysis of two different CNp doses using advanced spermatological analyses. However, certain points must be taken into consideration when interpreting the current findings. Although many structural and functional sperm parameters were comprehensively evaluated, the direct quantification of oxidative stress biomarkers was beyond the scope of the current experimental design. Therefore, the antioxidant effect of CNp has been interpreted in the context of functional outcomes and the existing literature. The inclusion of biochemical oxidative markers in future studies may further clarify the mechanism. Additionally, the lack of evaluation of size-dependent toxicity of CNps constitutes another limitation of the study. Furthermore, the results were obtained under controlled *in vitro* cryopreservation conditions. Although a comprehensive assessment of motility, mitochondrial function, membrane integrity, and DNA status provides strong biological evidence, fertility trials will contribute to confirming the field-validated cyclical validity of these findings.

This study, in its current form, is the first to conduct preliminary and detailed spermatological analyses for the cryopreservation of Angora buck semen and will serve as a fundamental resource for subsequent studies.

## 5 Conclusion

In conclusion, the present study demonstrates that curcumin nanoparticles exert measurable size- and dose-dependent effects during the cryopreservation of Angora buck sperm. Among the tested formulations, 20 nm CNp at 1 μg/ml provided the most consistent improvement in motility and preservation of DNA integrity, whereas 80 nm particles were associated with alterations in mitochondrial membrane potential. These findings underscore the critical role of nanoparticle physicochemical characteristics, particularly particle size, in modulating post-thaw sperm functionality. Within standardized cryopreservation conditions, CNp supplementation exhibited biological potential as a supportive strategy for improving buck semen quality. Further mechanistic investigations and fertility-based validations will be essential to optimize application parameters and confirm reproductive applicability under practical breeding conditions.

## Data Availability

The raw data supporting the conclusions of this article will be made available by the authors, without undue reservation.
